# The Sharing Economy in China’s Aging Industry: Applications, Challenges, and Recommendations

**DOI:** 10.2196/27758

**Published:** 2021-07-06

**Authors:** Yaolin Hu, Jian Wang, Stephen Nicholas, Elizabeth Maitland

**Affiliations:** 1 Dong Fureng Institute of Economic and Social Development Wuhan University Wuhan China; 2 Center for Health Economics and Management Economics and Management School Wuhan University Wuhan China; 3 Australian National Institute of Management and Commerce Sydney Australia; 4 Newcastle Business School University of Newcastle Newcastle Australia; 5 School of Economics and School of Management Tianjin Normal University Tianjin China; 6 Research Institute of International Strategies Guangdong University of Foreign Studies Guangzhou China; 7 School of Management University of Liverpool Liverpool United Kingdom

**Keywords:** sharing economy, aging industry, older adult population, older adults, aging, health services, sharing model, sharing, China, East Asia

## Abstract

**Background:**

All aging societies face the challenge of allocating limited resources for the highest value of use. The sharing economy provides one method to address the imbalance between the demand and supply of health services to the older adult population. With a substantial aging population, China’s practices in the sharing aging industry may set examples for other “getting old before getting rich” countries.

**Objective:**

There is a gap in both the data and research on China’s aging industry sharing economy. This paper addresses these data and research lacunae by constructing a framework for the application of a sharing model in China’s aging industry, by assessing the current state of the aging industry sharing economy, by setting out the challenges to the sharing aging health care and service economy, and by making recommendations for the development of the aging industry sharing economy.

**Methods:**

This paper constructs a sharing economy framework in the aging industry covering four aspects (*people*, *facilities*, *capital*, and *information*) to test the current state and future prospects of China’s aging industry sharing economy.

**Results:**

In people sharing, we analyzed the sharing of emotional companionship, doctors, nurses, nursing attendants, and domestic helpers. We discussed facility sharing models from the point of land and housing, medical devices, and other items such as pensioner meals and shared medicine bins. We acknowledge that crowdfunding platforms have developed fast in China, but many older adult users faced problems in their operation. Information sharing is a developing field, which can optimize users’ experiences and should help older adults filter out misinformation, but China currently does not have adequate sharing information platforms for older adults.

**Conclusions:**

We identified four major challenges in China’s aging industry sharing economy: poor adaptability to technology for older adults, mediocre quality of shared services, *one-size-fits-all* and the concept of the *useless elderly*, and shortage of qualified practitioners. We make recommendations for specific measures by governments, communities, and enterprises to improve the sharing economy in the aging industry.

## Introduction

### Population Aging

Population aging is a global phenomenon, with many aging societies such as western Europe, Japan, and the United States amassing experience in aging social governance. China is a typical resource-limited country that is “getting old before getting rich,” with 176 million or 12.6% of the population older than 65 years [1]. According to the United Nation’s criteria, China has been an aging country since 2000, with the older-aged population likely to exceed 300 million by 2025 as China transitions from a mildly aging country to a moderately aging one [2]. Only in 1994 did the Chinese government address the escalating population aging problem with a *Seven-year Development Program for China’s Work on Aging (1994-2000)*. The magnitude of the aging challenge is reflected in China’s gross domestic product (GDP) rank as the second in the world but its per capita GDP, estimated at US $10,261 in 2019, ranking only 80th in the world [3]. It is estimated that 600 million people in China earn only ¥1000 (about US $152) per month, with high poverty levels concentrated in the old aged [4]. China’s aging population has brought about complex social problems, including the demand for age care facilities and services, putting the government under severe governance and financial pressure.

### The Sharing Economy in the Aging Industry

#### Sharing Economy

Many problems brought about by population aging, such as shortage of health care staff, uneven distribution of aged care facilities, and poor services to the aged population, reflect the failure of age care resource supply to meet age care demand. Given the fixed and inflexible nature of many age care resources, the sharing economy’s innovations in technology and management promise to improve the balance between age care demand and supply. Faced by an absence of a consensus on the definition of the sharing economy and little published quantitative or qualitative research on the sharing aging industry [5,6], we specify an aging industry sharing economy framework, which is assessed and evaluated for China’s sharing aging industry. Organizations in the sharing economy have the following characteristics: they are organized as digital platforms enabling offline transactions between users, they facilitate peer-to-peer transactions and business-to-business operations, they emphasize temporary access rather than ownership, and they focus on the use of underused or undersupplied resources [7]. The sharing economy, as part of the digital economy [8], has transformed services, transport, finance, and education, with the health care industry starting to apply sharing models [9]. Sharing models match information on the supply and demand for resources with the help of internet technology. One of the most important features and influences of the sharing economy has been how it has changed people’s views on resources from ownership to the temporary right to access [10], which promotes the circulation of idle, misallocated, or underused resources and improves the efficiency of resource use. In 2019, the transaction volume of China’s sharing economy was estimated at ¥3.3 billion (US $509.5 million), comprising over 800 million citizens [11].

#### Aging Industry and Challenges to the Health Care Sharing Economy

The aging industry is a general name for all relevant industrial sectors that provide products and services to citizens in their old age and younger people preparing to enter old age, such as the wealth management and superannuation industries. China’s traditional culture of family-based old age care is being re-evaluated, as the middle-aged population consider their future old age care and their care of older-aged family members. Despite the aggressive innovation in digital platforms to manage the supply of products and services, provision of old age services remains unresolved in China. First, the development of the aging industry has not paid sufficient attention to the economic differences within the aging population in China. There has been a bifurcation between old age consumers of high-end health care products and services, not available to all, and low-end health care products consumed by less economically well-off older-aged consumers. Second, a *one-size-fits-all* approach has been adopted by most age health providers, with insufficient attention paid to the diverse physical and mental states of the older-aged consumer population, ignoring the differences between the younger elderly and older elderly. Third, there has been insufficient focus on resource supply shortages in the aging industry, especially nursing attendants, nursing homes, and other infrastructure.

The sharing economy promises the ability to maximize the value of underused and misallocated resources, and to address supply shortages, providing solutions to rebalance the supply and demand of aging industry resources. The Chinese government has started to address the underuse and undersupply of resources in the aging industry [12,13], supporting and guiding the development of the sharing economy with the aim to improve the efficiency of social resource use. In 2016, the *13th Five-year Plan for the Development of Civil Affairs* encouraged mutual assistance and coordinative efforts in the provision of old age service delivery. Although some enterprises in China’s aging industry have adopted the sharing and mutual assistance model, the sharing model in the medical treatment industry concentrates on serving people of all ages. Surprisingly, given the large size of the aged population, data and research on China’s aging industry sharing economy have been insufficient. We address these lacunae by constructing a framework for the application of the sharing model in China’s aging industry, by assessing the current state of the aging industry sharing economy, by setting out the challenges to the sharing aging health care economy, and by making recommendations for the development of the aging industry sharing economy.

## Methods

**Figure 1:**
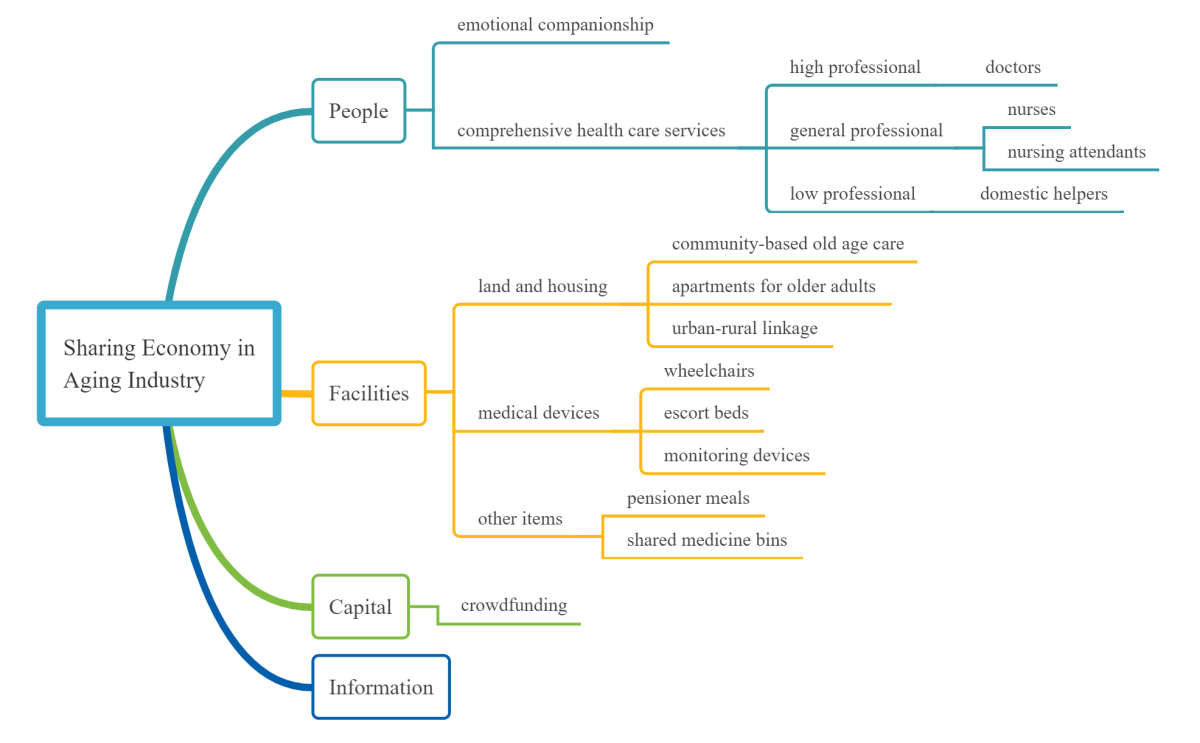
The framework of the sharing economy in the aging industry.

There has been little research on the sharing economy in the aging industry in China [6]. Depending on actual qualitative data from China’s sharing aging industry, our grounded method aims to explain the current status (the status quo) of an institution, China’s sharing aging industry, and its implications (challenges and applications). Following the grounded qualitative method, we collected and assembled data and information from a wide range of aging industry government, commercial and public sources and websites, academic papers, public reports, institutional reports, and fieldwork. The first analytical step was to categorize in Textbox 1 the types of services and activities applicable to the sharing economy model for older people. Second, we used the qualitative data in Textbox 1 to specify in Figure 1 an analytical framework for assessing the aging industry in China. Finally, we summarized, interpreted, and critically evaluated the information in Textbox 1 to explicate the sharing aging industry status quo and set out the challenges and implications, providing a range of recommendations.

In a 2016 study of sharing services for those 85 years or older, Ward and Coughlin identified meals, medication management, transportation, housekeeping, recreation and wellness activities, security, and personal care as elements in the sharing economy [14]. In China, the National Committee on Aging researched urban home-based older people’s satisfaction with domestic services, nursing services, chatting services, and legal aid services [15], finding these four aspects of old age services under substantial pressure. The Beijing Municipal Civil Affairs Bureau found that urban older people had little space for outdoor activities, but the opposite was true for rural older people [16]. Besides accepting services in their old age, older people were also willing to contribute to society in their old age. It was found that 45.6% of older adults (over 100 million people) often participate in various public welfare activities [17]. Based on government and public reports, websites, and academic papers, Textbox 1 categorizes the types of services and activities applicable to the sharing economy model for older people, both in terms of the status quo and prospective improvements for the future.

To conceptualize and operationalize the service categories in Textbox 1, Figure 1 provides a comprehensive sharing economy framework for the aging industry in China, comprising people, facilities, capital [9], and information.

In the next section, we assess the key elements in the aging industry sharing economy within a *people*, *facilities*, *capital*, and *information* framework in Figure 1.

**Services that could use the sharing economy model needed by older people from government, commercial and public welfare sources and websites, academic papers, public reports, institutional reports, and fieldwork.**

**Basic living needs**

- Houses
**Status quo**
  High urban housing prices make it difficult for children to house parents.
  Inadequate care for older parents due to children’s work requirements
  Most housing design does not cater for older adults’ needs
  The rural older adults live alone with large housing space because of young people’s immigration to cities, but the health care facilities are poor
  Some cities developing housing sharing services for older adults
**Prospect**
  Need for better home care
  Need for specialist housing for older adults
  The rural areas share housing resources with the urban areas
- Outdoor activity space
**Status quo**
  Scarce urban outdoor activity space for older adults
  Abundant rural outdoor space, but the activity facilities are poor
**Prospect**
  Communities can develop special activity space for older residents.
  By sharing idle land resources in rural areas, older adults in both urban and rural areas can get high quality activity space.
- Housekeeping
**Status quo**
  Sharing platforms for peer-to-peer housekeeping services need improvement.
**Prospect**
  Better sharing in housekeeping services can improve the life quality of home-based older people.
- Meals
**Status quo**
  Few specialist takeaway services catering to the health needs of older adults
  There have been sharing chefs to cook in users’ homes.
**Prospect**
  Need to expand sharing economy’s provision of nutritious meals suitable for older adults
- Transportation
**Status quo**
  Sharing transportation platforms in China (eg, Didi) do not cater for the travel needs of older adults.
**Prospect**
  Sharing transportation platforms for older adult

**Medical and nursing needs**

- Nursing care
**Status quo**
  Shortage of professional nursing services for older adults at home
**Prospect**
  Sharing model providing efficient allocation of nursing resources for older adults at home
- Medication management
**Status quo**
  Difficult for older adults to access limited high quality medical resources
  Older adults need web-based systems for reminder and renewal of medicine prescriptions.
**Prospect**
  Internet health care on access and adaptation
- Personal care
**Status quo**
  Good information for older adults on medical data
**Prospect**
  Internet medical care providing enhanced access to professional health service for older adults

**Emotional and spiritual needs**

- Recreation and chatting and company
**Status quo**
  Poor level of entertainment and spiritual needs of older adults
**Prospect**
  A sharing economy provides improved entertainment and spiritual companionship for older adults.
- Work
**Status quo**
  Many older people still want to participate in work.
**Prospect**
  Develop more sharing platforms to help older adults get job information.

## Results

### People

In the sharing economy model in Figure 1, sharing *people* refers to the supply side provision of people’s time, expertise, skills, or emotional company to old age consumers. Many old people taking care of themselves at home face the challenges of self-care ability, loneliness, and a feeling of being neglected [18]. Family planning, including the one-child policy and economic pressures on family size, have constrained the size of the average Chinese family to 3.1 persons in 2010, limiting family care for older family members [1]. In addition, the heavy influence of the Confucian tradition of *filial piety* is eroding among young people, especially those who have migrated from rural to urban areas, have less financial capacity to house, and have less time to meet the physical and emotional needs of old age family members. As shown in Figure 1, sharing of people can be divided into pure emotional companionship and comprehensive health care services, comprising high professional level, general professional level, and low professional level.

China has innovated in sharing people for emotional companionship, such as the *sharing children* model in Lianhu District, Xi'an City and the *caring tenants* project in Wuhan City. Supported by the community and funded by public welfare funds, Lianhu’s 2019 *good-neighbor sharing children* project recruited college students, with the consent of the old age person’s family, to share older persons’ extra rooms at a low price or free of charge. College students provided companionship, life care, and other services. Wuhan’s *caring tenants* project is similar, providing young people free-of-charge house sharing in return for daily companionship, with an adaptation period set before signing the mutual assistance agreement. In both projects, college students or young adults living with the old aged did not provide professional services but provided social engagement [19] and served the older aged emotional needs through companionship in their daily life.

Comprehensive health services relied on the expertise and skills provided by professional health specialists, divided into high professional, general professional, and low professional levels (Figure 1). The shared service suppliers at high professional level were mainly doctors, whose shared services in China has mainly been through telemedicine. Shared telemedicine services address the shortages of graded diagnosis and treatment, which are mainly provided at tertiary high-level hospitals. Older patients, especially those without accompanying family members, usually have poor access to timely tertiary hospital medical services, both due to their poor health constraining visits to a tertiary hospital and because their age and education level constrain their understanding of treatment choices between different hospital levels. Older patients with multimorbidities undertake many hospital visits, where many doctors prescribe only standardized, rather than personalized, chronic disease medicines [20]. Finally, hospital visits pose high risks of cross infections to older patients, especially those with low immunity to diseases. Telemedicine addresses these issues.

Telemedicine has been growing rapidly since 2000 in China, with internet medical platforms such as 39Health.com and DXY. Initially, user activity was not high, and the platforms were subject to restrictive policy controls. Currently, there are more than 900 internet hospitals in China, coupled with a telemedicine collaboration network covering more than 24,000 medical institutions in prefecture-level cities and more than 5500 hospitals at the secondary level or above that are capable of online services [21]. The COVID-19 outbreak has made hospitals more aware that telemedicine can help avoid cross infection, promote graded diagnosis and treatment, and make medical resources more accessible. During COVID-19, government authorities issued a number of official directions to accelerate the development of internet medical treatment. For example, the Wuhan Healthcare Security Administration launched medical insurance payment for the WeDoctor Internet General Hospital in early 2020, pioneering the formation of an online payment loop for diagnosis and treatment. By June 2020, the number of online medical users had reached 276 million, accounting for 29.4% of China’s total internet users [22]. In addition, during COVID-19, internet users purchased 26.4% of their medicine, health apparatus, and other medical supplies online, and 17.9% of the users accessed online medical services such as online registration and consultation [22]. Internet diagnosis and treatment apps allowed users to choose doctors according to their diseases, and doctors used texts, audio, and videos to diagnose online patients and prescribe treatments and medicine.

One example of an internet diagnosis and treatment app, Good Doctor covers 9852 public hospitals in China [23], with many well-known doctors accessed online when face-to-face appointments were hard to make. Importantly, apps such as Good Doctor do not require older-aged patients to have particularly high mobile phone operation skills, with these platforms providing voice input, voice recognition, and online phone calls for old age patients who cannot type texts on their mobile phones. Since the introduction of the *family doctor* policy in China, most of the online health platforms provide the option of signing a long-term contract for online consultation, prescription, and health consultation.

Doctor sharing models also include the combination of medical treatment and old age care, which benefit residents of old age care facilities [24]. In China, every thousand people had only 2.8 doctors (including assistant doctors) and 3.18 nurses in 2019 [25]. There were only 31.6 nursing beds per 1000 older-aged persons, but the nursing bed vacancy rate in some institutions was 50% to 60% [26]. Doctor sharing allows underused resources in old age care facilities to accept semidisabled or completely disabled older-aged people who are seriously ill. For example, to avoid older patients crowding out medical resources by using hospitals as nursing homes, doctor sharing models promote the combination of medical treatment and old age care within nursing homes and improve referral and cooperation between hospitals and nursing homes, maximizing both care and bed capacity. To promote doctor sharing, China’s National Health Commission and the World Health Organization launched 199 best practice examples of combining medical treatment and age care in 2019 [27]. One example is Beijing Longfu Hospital that combines apartments for older adults as a branch of the hospital, housing consulting rooms, and long-term doctor and nurse services to more than 200 old age apartment residents. In addition, the Longfu Hospital cooperated with external old age care institutions through online appointments and treatments.

In Figure 1, service providers at the general professional level include nurses and nursing attendants. In the doctor sharing model previously discussed, nurses and doctors work together to provide medical services to the old aged. The most typical model of nurse sharing in China is to reserve a nurse’s “door-to-door service” through an app. Existing nurse sharing apps such as Homeincare, U-nursing, and Goldnurse provide nurse services such as catheterization, bedsore nursing, stoma care, enema care, and sputum aspiration as well as companionship during consultations. The design concept for nursing sharing apps is similar to Uber, allowing nurses to take appointments outside the regular 9 to 5 working hours and allowing users to find suitable nurses who can provide door-to-door services based on their previous service evaluation results.

Although nursing assistants also provide everyday life care services for the old aged, there are constraints on the emergence and use of nurse attendants sharing models. First, there is a severe shortage of nursing attendants. In Beijing, for example, there are about 3.92 million people 60 years or older with Beijing household registration but only 15,000 nursing attendants [28]. Second, nursing attendants vary in competency. An access system for nursing attendants to serve old age patients was cancelled in 2017, with the government requiring the nursing institutions to strengthen nurse attendant training. Third, the social recognition of nursing attendants is not high, with many urban families hiring a nanny to undertake the nursing attendant role, and many rural families regard nursing attendants as an unnecessary luxury. Internet platforms have not addressed the imperfect nursing attendant market in China, which leaves many vulnerable older people without low-level daily care.

Service providers at the lowest professional level in Figure 1 are mainly domestic helpers, who undertake housework, cooking, and shopping for the old aged. Driven by internet technology, China’s domestic service market has formed a relatively mature online-to-offline (O2O) model, allowing users to make appointments and payments, leave comments, and rate domestic helpers through apps. One of China’s earliest O2O platforms, 58.com app, provides point-to-point housekeeping service information, optimizing the allocation of the domestic service labor force. China has also experimented with *time bank* platforms, where service providers do not get paid for their services but deposit in a time bank their service time, which they can use to pay for services they consume. One example is Crown Community of Yanliang District that organized appointments with workers willing to provide housekeeping services for the older adults through mobile phones. The service providers recorded their service time in the community’s time bank, which they could *withdraw* when they, or their older relatives, required help. In 2018, the Ministry of Civil Affairs of China explicitly included *time bank* in the nationwide pilot reform of home-based community old age care services, hoping to establish an operational model that could be promoted throughout the country [29]. So far, the time bank model has not been widespread in China.

### Facilities

Sharing of facilities is the primary objective in the sharing economy, with Airbnb and Uber the best-known examples. In China, sharing facilities for the old aged include land and housing, medical devices, and other items. In Figure 1, China’s aging industry can be divided into community-based old age care, apartments for older adults, and urban-rural linkage from the point of land and housing.

Community-based old age care is an extension and supplement to home-based care. Since the 1980s, communities have become the most important basic unit of social governance in China, which played a central role in the prevention and control of COVID-19 in 2020. Community-based old age care services are available in most cities, typically involving a shared space at day care centers. Although not sharing land and buildings, virtual nursing homes have been established in communities boasting good communications infrastructure, where the virtual community relies on a unified information management platform to meet older adult care needs online. For example, the Home-based Elderly Care Center in Suzhou is a virtual communications platform providing nursing home information through a *Fun-at-Home* older adult care service system, comprising 53 services in 6 categories, including housekeeping, property maintenance, and health care, such as companionship in hospital, entertainment and learning, and emergency help. Services are offered by professional management companies, and the platform allows older age users to order and evaluate services over the phone. The community plays an important role as a resource coordinator in both the establishment of day care centers and the virtual nursing home based on information technology. By integrating the community’s public space and older adult care service resources, community-based older adult care enables older adults to enjoy the benefits of home-based and professional older adult care at the same time.

Apartments for older adults are not nursing homes. Based on the experience of countries with apartments for the old aged in the United States, Japan, and Canada, apartments for the old aged provide an organizing body mainly for social functions, rather than social governance, and operate according to market rules. The construction of older adult apartments in China is currently showing uneven development. Although high-end apartments for older adults are in sufficient supply, due to high prices and low demand, low-end apartments, mainly funded by government financial aid and serving disadvantaged people, are in short supply. China is currently promoting the construction of low-end apartments for older adults and applying the concept of the sharing economy in the process. First, in terms of construction funds, the government mainly cooperates with private capital through the private-public partnership (PPP) model, rather than relying solely on government financial funds. Second, apartments for older adults are generally built in areas where living space is scarce or older adult care resources are insufficient. Third, jointly built by the government and private capital, low-end apartments for older adults often prompt hospitals in the vicinity to integrate their medical services with apartment-based older adult care. Government’s participation means that the price is relatively low, with Beijing’s first PPP apartment for older adults charging a housing fee plus basic service fee of only ¥4000 to ¥8000 (US $618-$1237) per month versus the commercial rate of ¥6000 to ¥16,000 (US $927-$2473) [30]. Through the integration of older adult care resources, this type of apartment improves the quality of older adult care services while also alleviating the financial pressure on older adults.

Urban-rural linkages in Figure 1 impact the old age sharing economy through rural-urban migration, a large rural aged population, poor rural old age care infrastructure including hospitals, and a persistent urban-rural income gap. Older adults in cities enjoy access to better medical resources and older adult care services than rural older adults, but urban areas boast less land and housing space for old age care facilities. According to a survey by the Beijing Municipal Civil Affairs Bureau on the home-based older adult care facilities in the urban area of Beijing, 11.8% of the registered communities had no outdoor activity space, 28.5% had only one outdoor activity site, and 23.6% had two or more outdoor activity sites [16]. Differences within cities and between urban-rural communities in land and residential space shapes the sharing economy. For example, more than 2000 farms in the Beijing municipality have joined a rural-urban sharing program, allowing farmers to post information about their idle land and houses on an app that other citizens can rent. Through farm sharing, not only can farmers increase their income, but older adults who have been living in cities for a long time and yearn for a rural life can expand their living experience. In Nanjing, to address the shortage of rural older adult care resources, professional nursing institutions have transformed idle homes of farmers into apartments for older adults and provided medical and nursing services at the same time.

Under facilities in Figure 1, medical devices are part of the sharing economy, although not all medical devices can, or should, be shared. Medical devices such as wheelchairs and escort beds can be accessed through mobile phones in a manner similar to the bike sharing model, and monitoring equipment is shared under the direction of medical staff. However, the sustainability of sharing medical equipment is likely limited, since the average price of a manual wheelchair is about ¥300 (US $46), making them affordable for families with immobile patients who require long-term wheelchair use. The sustainability of sharing escort beds is also questionable. Foldable beds for accompanying family members are an economical and efficient alternative to an escort bed. In addition, many hospitals provide accommodation services for accompanying family members. The sharing of monitoring devices is more like a self-service, where hospitals provide weight scales and blood pressure monitors at the hospital entrance for free use.

Additionally, under facilities in Figure 1, other items for older adult’s daily use are different from these items used by other age groups, such as food, which impacts China’s high rate of cardiovascular disease and cancer [31]. As previously discussed, the people sharing model allows older age people to make appointments for domestic helpers to cook home meals. Although China’s takeaway market focuses on younger people’s demand for fast food, there is scope for expanded *pensioner meals*, already provided by some restaurants, and for specialist takeaway meal providers to exclusively provide food for older adults. The Guangzhou government launched a program to deliver nutritious meals to older adults in 2016 [32], but the takeaway food market for the older aged requires further development. There are also examples of drug sharing models in China. *Shared medicine bins*, which work much like the vending machines, can be found in some communities, which dispense common medicines such as cold medicine, analgesic, and anti-inflammatory drugs. Users can pay at the shared medicine bin using their mobile phones.

### Capital

With more than 95% of the population covered by medical insurance since 2013, general health coverage is not a major issue requiring help from the sharing economy [33]. However, the small percentage of the noninsured population can use crowdfunding platforms, such as Shuidichou, Qingsongchou, and Aixinchou. For example, Shuidichou, founded in 2016, has raised more than ¥20 billion (US $3.09 billion) for patients with serious diseases from more than 250 million donors. These crowdfunding platforms are currently facing development difficulties in China. Although these crowdfunding platforms can fund people in financial need, they also face issues in their operation, such as false information, unregulated funds, and advertising harassment, with most crowdfunding platforms operated by profit-oriented companies.

### Information Sharing

In Figure 1, information sharing, also called aggregation models in China, is the final element in our sharing aging industry model. Aggregation models refer to the simultaneous access to many other platforms via one platform, where information sharing is not limited to a single platform. In 2017, the navigation software AMAP took the lead in connecting links of different bicycle and car sharing providers on its navigation page, and WeChat now provides free access to takeaways, sharing bikes and cars, sharing portable chargers, and secondhand item transfers. In aggregation models, the information sharing platform can expand the scope of service providers for its users and obtain more user data at the same time. Super platforms mean that sharing service providers can gain opportunities to acquire customers and increase market share at a lower cost. Users of the information sharing platforms can also gain knowledge of the quality and price of sharing services in the market and make choices more efficiently.

Although information sharing platforms such as travel platforms and car and bike sharing apps are widespread, information sharing models for older adults remain underdeveloped. Aggregation platforms can optimize users’ experiences, particularly when older age people face obstacles in using smart devices. An information sharing platform suitable for older adults should help older adults filter out misinformation. On Facebook, the amount of fake news shared by users older than 65 years is almost seven times that of the youngest group [34]. According to research on the internet life of older adults in China, the proportion of middle-aged and older people who have experienced internet fraud accounts was 67.3% of the interviewed population [35]. Information sharing platforms for older adults should have the facility to block false sharing services and to provide qualified sharing service options. Information sharing platforms should also provide safe and unified payment systems to reduce worries about mobile payments and reduce fraud, which will significantly affect the older adult’s use of mobile payment systems [36]. For convenient use, the design of internet products for older adults should be straightforward to use, such as the KOMP one-click computer that does not require any username and password like many other apps. KOMP’s main function is to help older adults to share photos and information, and to make video calls with family or friends [37].

Although China currently does not have a sharing information platform for older adults, some telemedicine firms have begun to realize the value of such aggregating models. For example, Ping An Good Doctor, DingDang Kuaiyao, WeDoctor, and other well-known internet medical service providers in China are providing professional information searches, online diagnoses and treatment, online pharmacies, and even commercial insurance information on their platforms.

## Discussion

### Strategies and Recommendations

Following the Result section, we identify four challenges and make recommendations for improvements to China’s sharing economy for the aging industry.

#### Promoting the Adaptability of Older Adults to Technology

Although China has 940 million internet users and a penetration rate of internet technology of 67% [22], the proportion of Chinese older adult internet users is far lower than that in many countries with more resources. Studies on the use of the internet by older people show that the familiarity with technology varies widely among older adults, depending on income, education, and social status [38]. Access to technology use among older adults not only makes social services more accessible but also enhances user’s happiness and social connectedness [39]. The outbreak of COVID-19 has reinforced the importance of internet technology for old people by emphasizing the importance of the internet, big data, and online health care in the prevention and control of the pandemic. The application of technology in the fight against the COVID-19 virus showed the weakness of the older adult group in internet technology acquisition [40]. For example, data platforms on citizens’ travel history, contacts, and disease history, which allowed citizens without COVID-19 symptoms to get a QR (Quick Response) *health code*, specifying their health status and travel rights, had been taken up by the young much more than older people. The government has identified the need to help older adults overcome obstacles to using technology [41,42], but the government, private enterprises, and community organizations should implement support to help older adults overcome obstacles in using technology, especially smartphones and the internet. Current mobile phones on the market mainly consider the needs of young people, but when designing products and providing services for older adults, enterprises should take into consideration smartphones with large screens, large fonts, long battery capacity, and simple operation. The government has been encouraging some companies to pay attention to older adult–friendly phones and list qualified products in a promotion catalog for older adults. In addition, internet service providers should consider developing models or pages suitable for older adults, providing more functions such as content reading, operation prompts, and voice assistance. Community workers could also help older adults use smart technology and the internet, for example, through community-based courses for older adults to learn to use smartphones and the internet. In a country that highly values filial piety, young people should help their elders to bridge the technological gap. At the same time, the government needs to protect the retention of traditional service methods familiar to older adults, such as retaining cash payments.

#### Improve the Level of Shared Services for Older Adults

There are three development problems to overcome to improve China’s shared older adult care industry: insufficient development in scope, imbalance of the industrial structure, and compliance issues. Insufficient development in scope means an inadequate spread of many shared older adult care services. The *doctor sharing* model is a success story, especially during the COVID-19 pandemic, which has proved the feasibility of the people sharing model and verified both its business value and social contribution. Other shared services such as nursing attendants have failed to provide widespread resources, and there is no qualified nursing attendants information sharing platform for older adults.

Additionally, the imbalance of industrial structure reflects the differential access to shared services by region (the industrial east and coastal provinces vs underdeveloped and western regions), by urban-rural and by rich and poor older adult households. Since shared older adult market-driven service models have quasi-public characteristics, there is a need for government to engage private entrepreneurs and firms in the older adult service market to expand older adults’ access to resources. Long-term inequalities by region require national government interventions. Finally, by compliance issues, we mean sharing economy providers who prioritize attributes such as the speed and scale of expansion over platform compliance to the needs of older adults. For instance, shared nursing attendants services resulted in cases of abuse of older adults, and fundraising platforms marketing financial assistance sharing services have been subject to scams.

We recommend state interventions to address these three challenges. First, we suggest the central government should formulate guiding policies to promote the development of the shared aging care and service industry. Specifically, we recommend that the central government strengthen special research on shared aging and provide more comprehensive and specific support policies. These guiding policies should cover multiple fields such as human resources, older adult care facilities, medical services, and industry development. In addition, we recommend subnational governments at all levels should also formulate corresponding policies to promote the development of the local shared older adult care industry based on the endowment of local resources.

Second, the central government should finance an appropriate supply of shared older adult care services, especially to older adults unable to pay for shared services. Central and local governments should expand PPPs, especially in the construction of low rent apartments for the financially less well-off older adults. Changes in land use regulations should be implemented to integrate land, housing, and medical resources in the sharing model. For private enterprises participating in shared older adult care, the government should extend tax incentives.

Third, the state should improve the supervision mechanism on shared older adult care services and service providers [43]. All shared older adult care platforms should have service complaint or feedback channels to protect the rights and interests of older adults, based on industrywide standards. At present, many shared older adult care service platforms in China do not provide feedback. At the same time, these complaint and feedback channels need to be designed to suit the operating habits of older adults, like providing more voice prompts and direct telephone calls, and avoid the use of inflexible artificial intelligence responses. Local, provincial, and national regulators should exercise external supervision, improving the legal provisions to address compliance issues. Based on the framework of Figure 1, the most urgent supervisory need is to strengthen legislative supervision of crowdfunding activities controlled by for-profit companies [44].

#### The “One-Size-Fits-All” and the Idea of “Useless Elderly”

One problem in the provision of shared services for older adults is the lack of awareness of diversity among older-aged people. Although some older-aged people experience more chronic diseases, multimorbidities, and declining mental acuity [45], many old people reject a life of *doing nothing*. However, many old aged shared service providers view older adults as nonproductive or the *useless elderly*. For example, many apartments for older adults do not provide facilities and venues for creative activities. State regulations should require the provision of social and active centers at community-based old age care facilities and apartments, which can reduce the risk of ill health and improve happiness in old age [46,47]. Existing service providers of shared older adult care should address the spiritual needs of different types of older adult people and provide more opportunities for older adults to participate in society. According to the fourth sampling survey of the living conditions of older adults in China’s urban and rural areas, 72.9% of older adults were willing to help other older adults with difficulties in the community, and 21.4% of older adults had made suggestions to the community [48]. Rather than the *useless elderly*, various large cities in China have recruited older people to volunteer for public services, such as mediating neighborhood disputes and serving as information helpers at the city’s subway stations, bus stops, and tourist sites. We recommend that the government set up information sharing platforms that mainly publish city volunteer work information for older adults. Private enterprises should be encouraged to build information sharing platforms for older adult job seekers and recruitment companies so that older adults also have the opportunity to obtain labor income after retirement.

#### Shortage of Qualified Practitioners

An important practical reason to develop shared older adult care is the shortage of aging industry practitioners, including doctors, nurses, and nursing attendants. It is estimated that there was a shortage of 9.3 million trained nursing attendants [49], and existing doctors and nurses face high work pressure, long work hours, and low salaries, especially in public hospitals. The sharing model can facilitate the flow of information to identify high-quality health workers and to improve users’ evaluation of the medical and health industry, which will allow more efficient allocation of human health care resources. Additionally, with the help of sharing platforms, health workers can serve more customers, efficiently allocate scarce human resources, and allow health practitioners to obtain higher incomes. Finally, sharing models, subject to public supervision, can promote a more standardized service to older adults, improve the working environment for health workers, and encourage new recruits to enter the health care industry.

Although the sharing economy model can help solve the problem of the shortage of health workers, government financial resources still need to be allocated to address the shortages of health workers, including doctors, nurses, and nursing attendants. First, the state should further guarantee doctor and nurse incomes, including incentive salaries for doctors and nurses. Among China’s 98 industry classifications, the salary of health workers ranks 26th in the country, which is only 1.2 times the average salary in China [50]. Sharing platforms can provide incentives for doctors and nurses to earn additional income through multisite practicing and incentive payments for additional work.

Second, we recommend addressing unprotected health workers’ labor rights, especially irregular labor contracts and the absence of guaranteed social insurance, where nursing attendants are especially disadvantaged. For example, the work intensity of nursing attendants is high, with a nursing attendant in a nursing home taking care of 6 to 7 older people [51], and the salary of nursing attendants are often lower than that of domestic workers. Finally, unified professional training and consistent regulations are required for professional health workers, which will help address the shortages of nurses and nursing attendants.

### Conclusion

Constructing a sharing economy framework, comprising people, facilities, capital, and information, we analyzed the current status and prospects for the future development in China’s sharing aging industry economy. In people sharing, we identified shortfalls in the sharing of emotional companionship, doctors, nurses, nursing attendants, and domestic helpers. Facility sharing models required improvements in the allocation of land and housing, medical devices, and other items like pensioner meals and shared medicine bins. Although crowdfunding platforms have developed fast in China, difficulties in shared capital platforms opened them to compliance issues. Information sharing is a developing field, which can optimize older users’ knowledge needs and help filter out misinformation, but sharing information platforms for older adults remain both underdeveloped and unregulated.

From the analyses of the key elements in the sharing economy model, we identified four major challenges for older adults: poor adaptability to technology for older adults, mediocre quality of shared services, *one-size-fits-all* and the concept of the *useless elderly*, and the shortage of qualified practitioners. To address these gaps, we recommended specific actions by the government, communities, and enterprises. To promote the adaptability of technology for older adults, older adults need support and training to overcome obstacles in using technology, especially smartphones; enterprises should design internet products and services for older adult’s convenience; and communities, especially community workers and young people, should aid older adults in smart technology and internet use. Since shared older adult market-driven service models have quasi-public characteristics, we recommend state interventions. The mindset that *one-size-fits-all* and the idea of *useless elderly* must change. The state should legislate for social and active centers at community-based old age care facilities and apartments, and the state should encourage, and enterprises provide, platforms for older adult job seekers and volunteer workers. To promote the shared health aging industry, the state should expand the number of qualified practitioners, guarantee doctor and nurse incomes, address irregular labor contracts, especially for nursing attendants, and create unified professional training to alleviate the shortages of doctors, nurses, and nursing attendants. Our analysis of China’s sharing economy in the aging industry will be instructive for other countries *getting old before getting rich*.

## Abbreviations

GDP: gross domestic product
O2O: online-to-offline
PPP: private-public partnership
QR: Quick Response
